# Land use and Season Drive Compositional Shifts in Cyanobacteria-dominated Soil Bacterial Communities

**DOI:** 10.1007/s00248-026-02759-6

**Published:** 2026-04-23

**Authors:** Lisa Signorile, Mohammad Yaghoubi Khanghahi, Francesco Maria Calabrese, Carmine Crecchio, Rosangela Addesso, Margherita Chiarini, Adriano Sofo

**Affiliations:** 1 Independent Researcher, National Geographic, Rome, Italy; 2 Department of Agricultural, Forestry, Food and Environmental Sciences (DAFE), Università degli Studi della Basilicata, Via dell’Ateneo Lucano 10, Potenza, 85100 Italy; 3 Department of Soil, Plant and Food Sciences, University of Bari “Aldo Moro”, Via Amendola 165/A, Bari, 70126 Italy

**Keywords:** Cyanobacteria–heterotroph interactions, Diazotrophic bacteria, Nitrogen-free enrichment, Soil microbial ecology

## Abstract

Cyanobacteria-dominated bacterial communities in soils were investigated using a selective cultivation approach to evaluate the effects of land use and season. Soils were sampled from cropland and non-agricultural terrestrial habitats in spring and autumn, and microbial growth was enriched on modified Bristol medium lacking inorganic nitrogen before being analyzed by 16 S rRNA gene sequencing. Alpha diversity did not differ significantly between land-use types or sampling seasons, indicating that functional selection imposed by cultivation constrained within-sample diversity. In contrast, community composition showed consistent differentiation across both factors. Multivariate analyses demonstrated that land-use- and season-related patterns were primarily driven by shifts in a limited number of cyanobacteria-associated heterotrophic bacterial genera. In contrast, filamentous cyanobacteria showed comparatively stable representation across environments and seasons. These results indicate that, within functionally selected soil microbial systems, environmental signals are more effectively captured by changes in associated bacterial partners than by variation in cyanobacterial diversity or dominance.

**Supplementary Information:**

The online version contains supplementary material available at 10.1007/s00248-026-02759-6.

## Introduction

Soil cyanobacteria are key contributors to terrestrial ecosystem functioning, particularly through their roles in primary production, nitrogen fixation, and soil surface stabilization. Beyond their direct ecological functions, cyanobacteria form tightly associated consortia with heterotrophic bacteria, giving rise to cyanobacteria-centered microbial communities that influence nutrient cycling and microscale biogeochemical processes in soils [1]. While the distribution and diversity of soil cyanobacteria have been investigated across a range of terrestrial environments, considerably less is known about how land use and seasonal variation shape the broader cyanobacteria-associated bacterial community.

High-throughput sequencing studies have revealed extensive taxonomic diversity across ecosystems; however, this diversity often masks the ecological interactions that structure microbial consortia and regulate ecosystem processes. Increasing evidence indicates that microbial communities operate as interconnected networks in which metabolic exchanges, resource partitioning, and cooperative interactions shape community assembly and function. In particular, phototrophic microorganisms, such as cyanobacteria, frequently form stable associations with heterotrophic bacteria that use cyanobacteria-derived organic compounds or participate in complementary metabolic pathways. These interactions regulate carbon and nitrogen cycling and influence the stability and resilience of microbial communities in soil environments [2, 3].

Environmental factors play a central role in structuring these microbial assemblages. Among them, land-use practices represent a major driver of soil microbial community composition and functional potential. Agricultural management practices such as tillage, fertilization, and crop rotation can modify soil structure, organic matter inputs, and nutrient availability, thereby influencing microbial activity and community composition. Long-term land-use changes have been shown to reshape bacterial assemblages and alter ecosystem functions related to nutrient cycling and soil fertility [4, 5]. At the same time, seasonal environmental variability can further influence microbial community dynamics. Seasonal fluctuations in soil temperature, moisture, and plant-derived carbon inputs often lead to recurrent shifts in microbial community composition and metabolic activity, particularly in Mediterranean ecosystems characterized by pronounced transitions between wet winters and warm, dry summers [6, 7].

Previous studies investigating soil microbial communities have relied on culture-independent sequencing approaches, which have greatly improved our understanding of microbial diversity across terrestrial ecosystems. However, such approaches often capture extremely complex communities in which functional interactions among specific microbial consortia remain difficult to resolve [8]. Selective cultivation provides a complementary framework by enriching functionally defined microbial assemblages and reducing background diversity. In particular, nitrogen-free enrichment media preferentially select for diazotrophic cyanobacteria while allowing the persistence of a subset of closely associated heterotrophic bacteria capable of utilizing cyanobacteria-derived metabolites. By simplifying community composition while maintaining ecologically relevant microbial partners, this approach can enhance the detection of environmental signals related to land use and seasonal variation within functionally constrained microbial systems shaped by nitrogen limitation [9, 10].

Land-use change and agricultural management practices are known to alter soil physicochemical properties and microbial community composition, whereas seasonal fluctuations in temperature, moisture, and light availability can influence microbial activity and interactions [11, 12]. However, it remains unclear whether these environmental gradients primarily affect cyanobacterial taxa themselves or are more strongly reflected in the composition of associated heterotrophic bacterial partners within cyanobacteria-dominated assemblages.

The objective of this study was to determine whether land use and seasonal variation structure cyanobacteria-dominated bacterial communities enriched under nitrogen-free conditions. Specifically, the study aimed to assess whether differences in land-use type (cropland soils versus non-agricultural terrestrial habitats) and sampling season (spring versus autumn) are reflected in changes in within-sample diversity (alpha diversity) or, alternatively, in between-sample compositional dissimilarity (beta diversity), reflecting shifts in the relative abundance structure of taxa within cyanobacteria-enriched assemblages. By distinguishing between diversity-based and composition-based responses, the study sought to evaluate whether environmental gradients primarily affect cyanobacterial taxa themselves or are more sensitively captured by associated heterotrophic bacterial partners, thereby providing insight into the ecological organization and environmental responsiveness of functionally selected soil microbial consortia.

## Materials and Methods

### Experimental Site and Soil Sampling

Soil samples were collected from ten distinct terrestrial locations representing two land-use categories: cropland soils and non-agricultural terrestrial habitats (hereafter referred to as NAT habitats). Cropland sites were located in Policoro (Basilicata region, Southern Italy; 40°10′20″ N, 16°39′04″E) and are part of a long-term field experiment comparing conventional, minimum, and no-tillage management under a biennial rotation of durum wheat (*Triticum durum* Desf.) and faba bean (*Vicia faba* L.). Two additional cropland sites consisted of an almond grove (Modugno, Apulia region, Southern Italy, 41°05′27″N, 16°45′21″E), subjected to different management with and without compost amendment.

NAT habitats encompassed a heterogeneous set of natural and semi-natural environments, including two cave environments: Grotta della Capra (Apulia region, Southern Italy; 40°52’39"N 16°35’32” E) and Grotta di San Michele (Apulia region, Southern Italy; 40°49′12″ N, 16°25′12″ E). Additional NAT sites comprised forest (Mercadante Forest), prairie (Alta Murgia National Park), and urban stone surface (city walls and pavements) habitats located in the Apulia region (southern Italy; see Supplementary Table S1 for site coordinates).

For each site, samples consisted of composite material obtained by pooling approximately 20 spatially distributed subsamples collected within the same habitat. Sampling was randomized, with soil and rock samples collected in a scattered manner across the study area. Sampling was conducted during two seasonal campaigns in 2024: spring sampling took place between 28 April and 4 May 2024, while autumn sampling was conducted between 10 and 17 October 2024. A total of 20 samples were collected (*n* = 10 per season and *n* = 10 per land-use category). Each site × season combination was treated as a single experimental unit and was subsequently analyzed to assess seasonal shifts.

### Preparation of Soil Suspensions and Selective Enrichment of Cyanobacteria

Soil suspensions were prepared by adding 10 g of fresh soil to 90 mL of distilled water containing 0.1 g sodium pyrophosphate, followed by shaking at 100 rpm for 10 min to obtain a 10^− 1^ (w/v) suspension. Serial dilutions (10^− 2^, 10^− 3^, and 10^− 4^) were subsequently prepared. Aliquots (1 mL) from each dilution were inoculated into 9 mL of a modified Bristol liquid medium lacking inorganic nitrogen and supplemented with vitamin B12, with ten replicate tubes prepared per dilution and treatment. The nitrogen-free medium imposed a selective pressure favoring nitrogen-fixing cyanobacteria and closely associated diazotrophic microorganisms, thereby limiting the proliferation of non-diazotrophic heterotrophs during cultivation. The medium composition (g L^− 1^) was as follows: CaCl₂ (0.025), MgSO₄·7 H₂O (0.075), K₂HPO₄ (0.075), KH₂PO₄ (0.018), NaCl (0.025), and FeCl₃ (0.005). Cultures were incubated for 30 days under control conditions (25 °C; 12 h light/12 h dark photoperiod) using full-spectrum LED lighting (18 W), allowing the development of cyanobacteria-dominated microbial assemblages.

### Microscopic Observation and Morphological Characterization of Cyanobacteria

For each composite soil sample, three tubes showing evident cyanobacterial growth, typically originating from the 10⁻² dilution, were selected for microscopic examination. Cultures were gently homogenized by shaking, and aliquots were mounted on glass slides, covered with coverslips, and immediately examined using a light microscope (ADL 601 P, Bresser GmbH, Rhede, Germany) equipped with a digital camera (MikroCam II, 20 MP). Observations were conducted under transmitted and polarized light using phase-contrast optics at 400× and 600× magnification. Morphological identification of filamentous and unicellular cyanobacteria followed the classical taxonomic criteria described by Rippka et al. [9] and later cyanobacterial classification frameworks [13]. Microscopic observations were used to verify the presence and morphological consistency of cyanobacterial growth in the enrichment cultures and to provide qualitative confirmation of cyanobacterial development during cultivation.

### DNA Extraction, 16 S RRNA Gene Library Preparation, Sequencing, and Bioinformatics Analyses

Bacterial community composition was analyzed using DNA extracted from cyanobacteria-enriched microbial assemblages obtained under nitrogen-free culture conditions. Total DNA was isolated from harvested biomass using the FastDNA™ SPIN Kit (MP Biomedicals, Santa Ana, CA, USA) for Soil following mechanical lysis and silica-based purification, and DNA quantity and integrity were assessed fluorometrically and electrophoretically. Sequencing libraries targeting the V3–V4 hypervariable regions of the bacterial 16 S rRNA gene were prepared according to standard Illumina protocols, including amplicon amplification, purification, dual indexing, and normalization. Equimolar libraries were pooled, spiked with 30% PhiX to increase sequence diversity, and sequenced on an Illumina MiSeq platform (Illumina Inc., San Diego, CA, USA) using 2 × 250 bp paired-end chemistry.

PCR primers and Illumina adapter sequences were trimmed using the Cutadapt plugin [14] implemented within the QIIME2 pipeline [15]. Sequence quality was evaluated using FastQC and summarized with MultiQC to assess base quality distribution and potential sequencing artifacts. Reads were subsequently denoised using the Deblur algorithm within QIIME2 [16], which performs error correction and generates amplicon sequence variants (ASVs) after filtering low-quality reads and removing potential sequencing errors. Taxonomic classification was performed using a naïve Bayes classifier trained on the SILVA ribosomal RNA database reference database (release 138), trimmed to the V3–V4 region amplified by the primer pair [17]. Before classifier training, reference sequences were filtered by removing low-quality entries, dereplicating identical sequences, and restricting the dataset based on sequence length and taxonomic annotations.

A total of approximately 1.4–1.6 million raw paired-end reads were obtained across all samples. Following quality filtering, denoising, and chimera removal within the QIIME2 pipeline, a reduced set of high-quality non-chimeric reads was retained for downstream analyses. The proportion of chimeric sequences was consistently low across samples (generally < 2%), indicating high sequence quality. Detailed sequencing statistics, including raw reads, post-denoising reads, and chimera counts for each sample, are provided in Supplementary Table S2.

Alpha diversity was calculated using the Shannon diversity index based on the relative abundance of amplicon sequence variants. Differences in alpha diversity between land-use categories and sampling seasons were evaluated using the non-parametric Mann–Whitney U test. Community composition (beta diversity) was assessed using Bray–Curtis dissimilarity matrices calculated from relative abundance data. Differences in community composition between groups were tested using permutational multivariate analysis of variance (PERMANOVA) with 999 permutations. Multivariate patterns of community structure were visualized using principal component analysis (PCA). In addition, Partial Least Squares Discriminant Analysis (PLS-DA) was applied as a supervised multivariate approach to identify bacterial genera that most strongly contributed to seasonal and land-use-related differentiation.

## Results

The nitrogen-free Bristol medium imposed a selective pressure favoring diazotrophic cyanobacteria and closely associated microorganisms while restricting the growth of many non-diazotrophic heterotrophs. Following incubation, community composition was assessed by 16 S rRNA gene amplicon sequencing. In most enrichment cultures, cyanobacteria represented more than 50% of total bacterial sequences (Fig. S1). Therefore, the resulting assemblages are hereafter referred to as cyanobacteria-dominated communities, acknowledging that this designation reflects relative sequence abundance after enrichment rather than direct quantification of cyanobacterial biomass in the original soil samples.

**Fig. 1:**
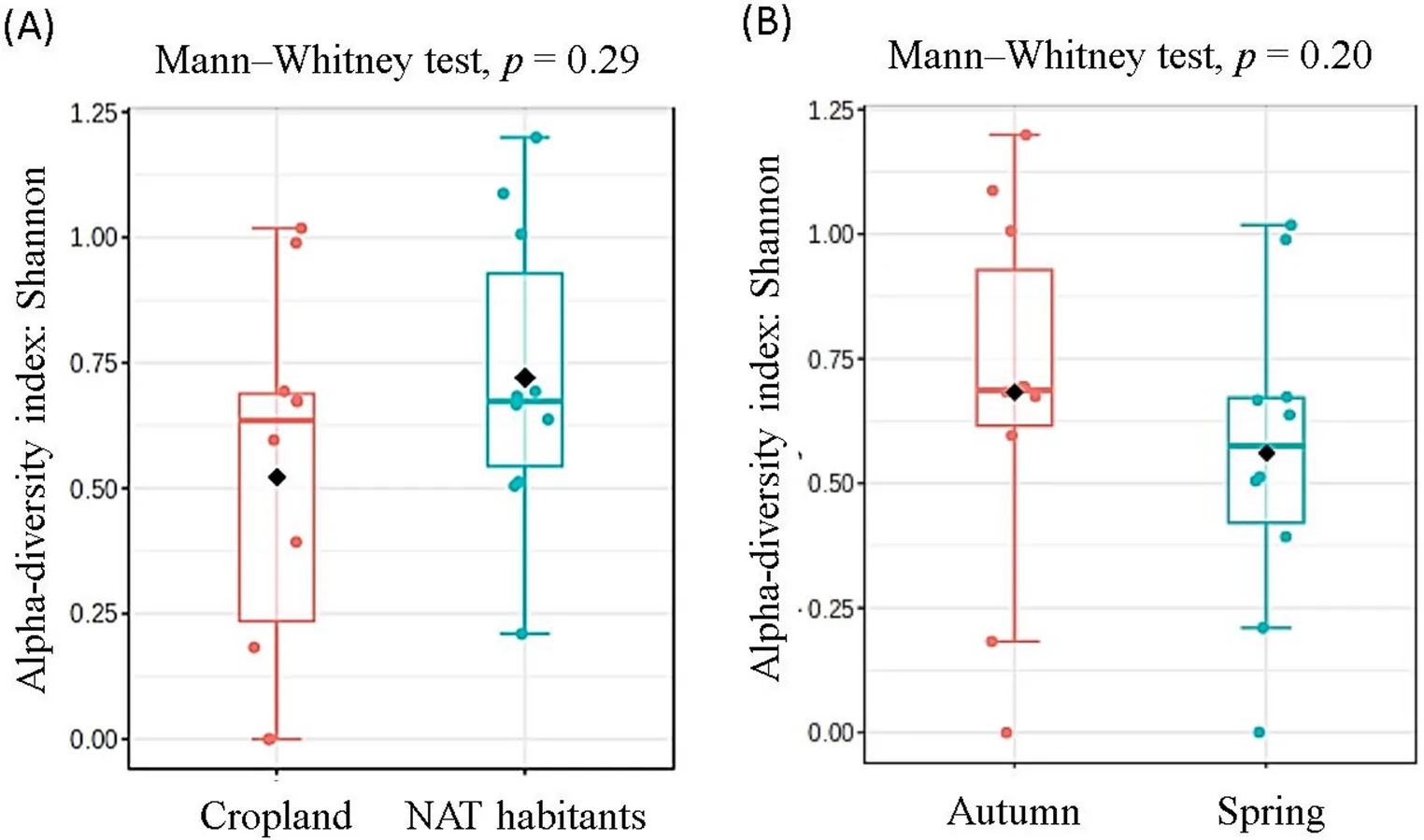
Alpha diversity of cyanobacteria-dominated bacterial assemblages enriched under nitrogen-free culture conditions, expressed as the Shannon diversity index. Assemblages originated from cropland soils and non-agricultural terrestrial (NAT) habitats and were sampled in spring and autumn. Each data point represents one site × season experimental unit. No significant differences in alpha diversity were detected between land-use categories or sampling seasons (Mann–Whitney tests, *p* ≥ 0.05)

Alpha diversity patterns were first evaluated to assess whether land use or season influenced within-sample diversity of cyanobacteria-dominated bacterial assemblages. Shannon diversity indices did not differ significantly between cropland soils and NAT habitats, nor between spring and autumn sampling periods (Fig. 1A, B), indicating comparable richness and evenness across land-use types and seasons.

**Fig. 2:**
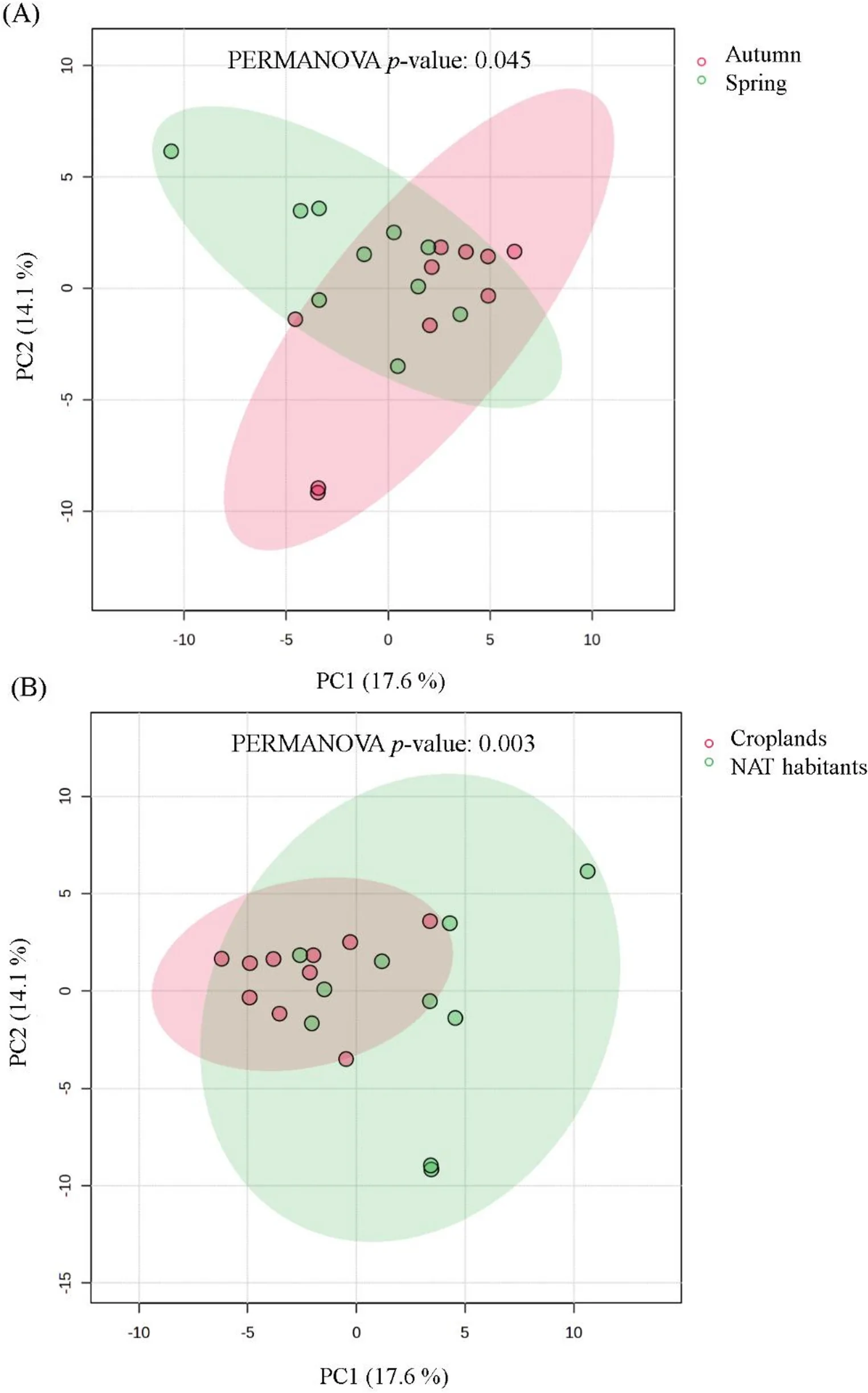
Principal component analysis (PCA) of cyanobacteria-dominated bacterial community composition based on 16 S rRNA gene amplicon sequencing, including ordination according to sampling season (spring vs. autumn) (**A**) and ordination according to land-use type (cropland soils vs. non-agricultural terrestrial habitats (NAT)) (**B**). Each point represents one site × season experimental unit. Ellipses indicate 95% confidence intervals. Differences in community composition were evaluated using PERMANOVA (999 permutations)

Despite this apparent stability in alpha diversity, multivariate analyses revealed clear differences in community composition. Principal component analysis showed consistent structuring of assemblages along both land-use and seasonal gradients. Samples originating from cropland soils and NAT habitats occupied partially distinct regions of ordination space (Fig. 2A). Spring and autumn samples also displayed pronounced compositional separation (Fig. 2B). These patterns, supported by PERMANOVA results, indicated that both land use and season contributed to the observed variation in community composition.

At the phylum level, when data were examined independently both for land use and season, distinct marginal patterns emerged. Averaged across seasons, cropland soils showed slightly higher cyanobacterial relative abundance (55.1%) than NAT habitats (50.7%). In contrast, seasonal differences were more pronounced when averaged across land-use categories, with cyanobacteria accounting for 62.4% of the enriched community in autumn but declining to 43.5% in spring, accompanied by a corresponding increase in other bacterial phyla (Fig. S1).

**Fig. 3:**
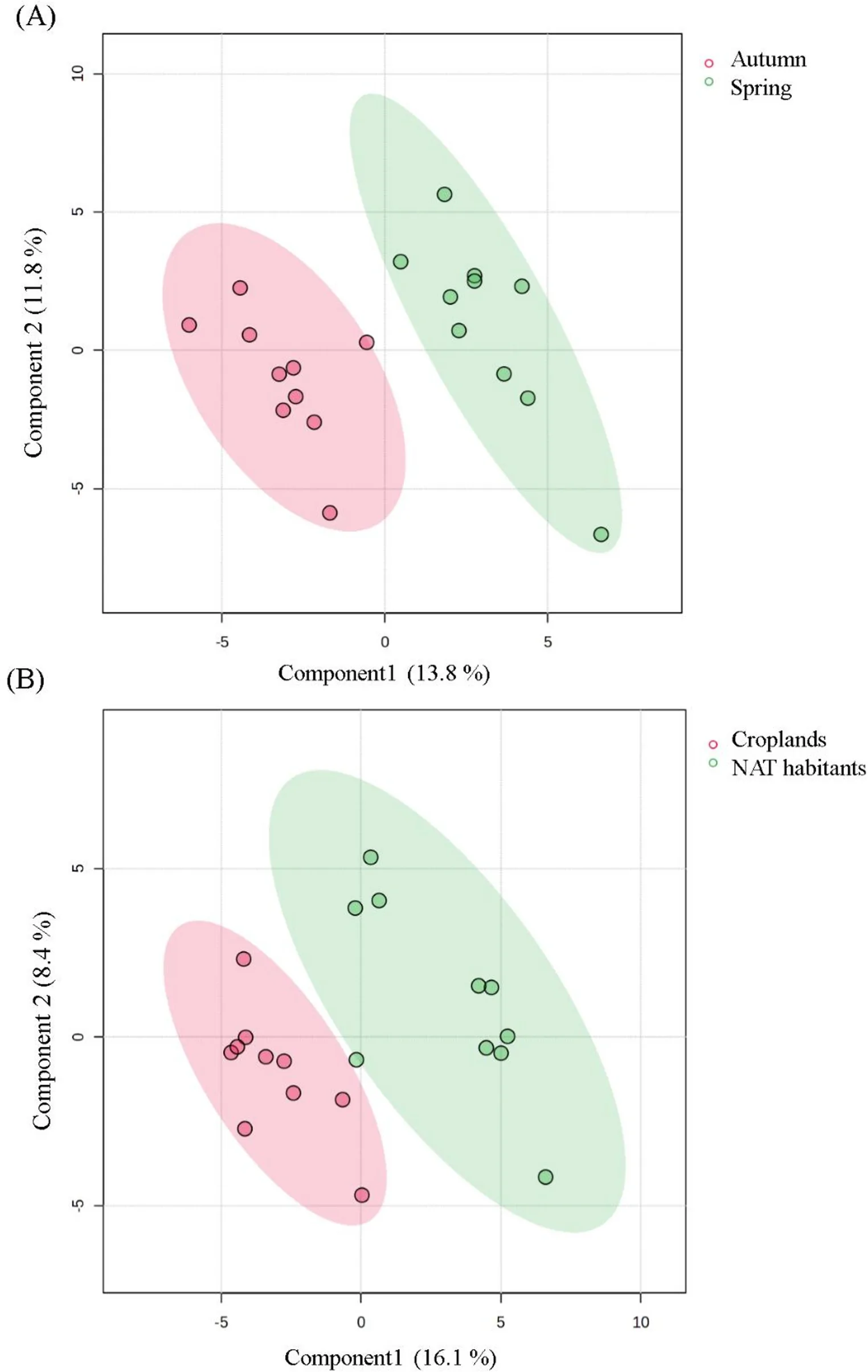
Partial Least Squares Discriminant Analysis (PLS-DA) of cyanobacteria-associated bacterial communities at the genus level, indicating compositional separation between spring and autumn samples (**A**) and cropland soils vs. non-agricultural terrestrial habitats (NAT) (**B**). Model robustness was evaluated using cross-validation (Q² > 0.6), indicating good predictive performance and reducing the likelihood of model overfitting

To better discriminate taxa underlying these compositional patterns, PLS-DA was performed at the genus level. The overall PLS-DA revealed structured differentiation among cyanobacteria-dominated bacterial communities along both seasonal and land-use gradients (Fig. 3). Model robustness was confirmed by cross-validation metrics (Q² > 0.6; Fig. S2). The parallel performance of seasonal and land-use models, combined with stable R² and Q² values, indicates that the observed separations were not driven by overfitting but reflected consistent compositional patterns captured by the PLS-DA framework. Although the first two ordination axes explained a modest proportion of the total variance, this is common in high-dimensional microbial datasets. The ordination nevertheless revealed consistent clustering patterns supported by PERMANOVA, indicating significant compositional differences between seasons and land-use categories.

**Fig. 4:**
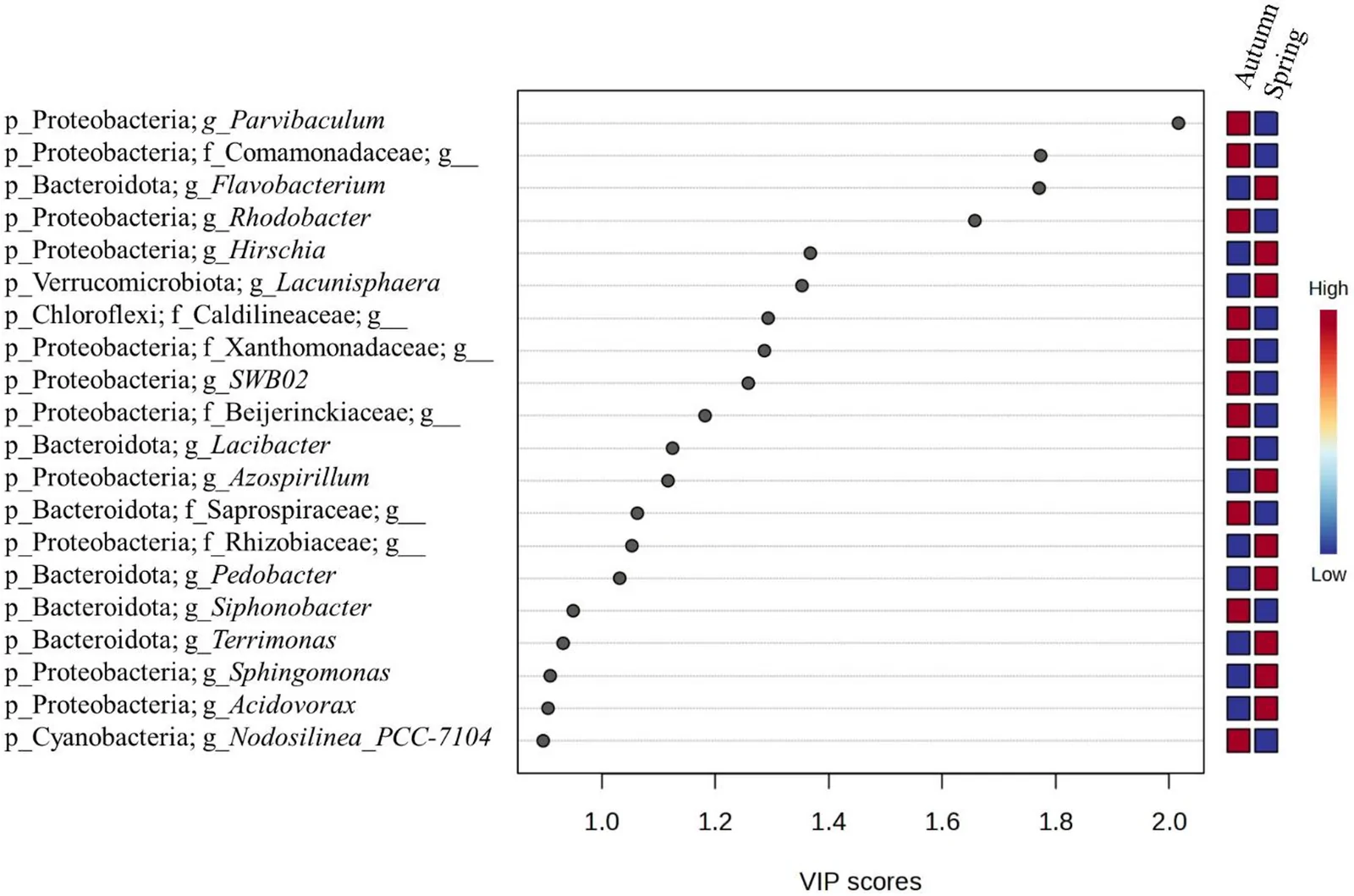
Partial Least Squares Discriminant Analysis (PLS-DA) of cyanobacteria-associated bacterial communities at the genus level, indicating compositional differentiation between spring and autumn samples. The model was optimized using 5-fold cross-validation based on the Q² performance metric, with a maximum of two components evaluated. The ordination is based on the 20 bacterial genera with the highest Variable Importance in Projection (VIP) scores, representing those taxa that contributed most strongly to seasonal discrimination. The heatmap represents relative representation patterns across treatments and is provided for visualization purposes; taxa were selected based on their VIP scores, indicating their contribution to group discrimination

To identify taxa contributing to seasonal differentiation, PLS-DA was applied at the genus level using the 20 taxa with the highest VIP scores (Fig. 4). The seasonal signal was not driven by a single taxon but emerged from the combined contribution of multiple phylogenetically distinct bacterial groups. Several genera affiliated with Bacteroidota, including *Flavobacterium*, *Lacibacter*, *Pedobacter*, *Siphonobacter*, and *Terrimonas*, were among the taxa contributing to the separation of spring-associated assemblages. The verrucomicrobial genus *Lacunisphaera* of spring taxa also contributed significantly to the seasonal separation. In contrast, several Proteobacteria, including *Parvibaculum*, *Rhodobacter*, *Hirschia*, *Azospirillum*, *Sphingomonas*, *Acidovorax*, and *SWB02*, as well as uncultured representatives of *Comamonadaceae*, *Rhizobiaceae*, *Xanthomonadaceae*, and *Beijerinckiaceae*, contributed more strongly to the differentiation of autumn-associated communities. Additional contributions were observed for the Cyanobacterial genus *Nodosilinea* and uncultured members of Caldilineaceae (Chloroflexi). The accompanying heatmap illustrates the relative representation of these taxa across seasons but should be interpreted as a visualization of discriminatory patterns rather than a direct test of differential abundance.

**Fig. 5:**
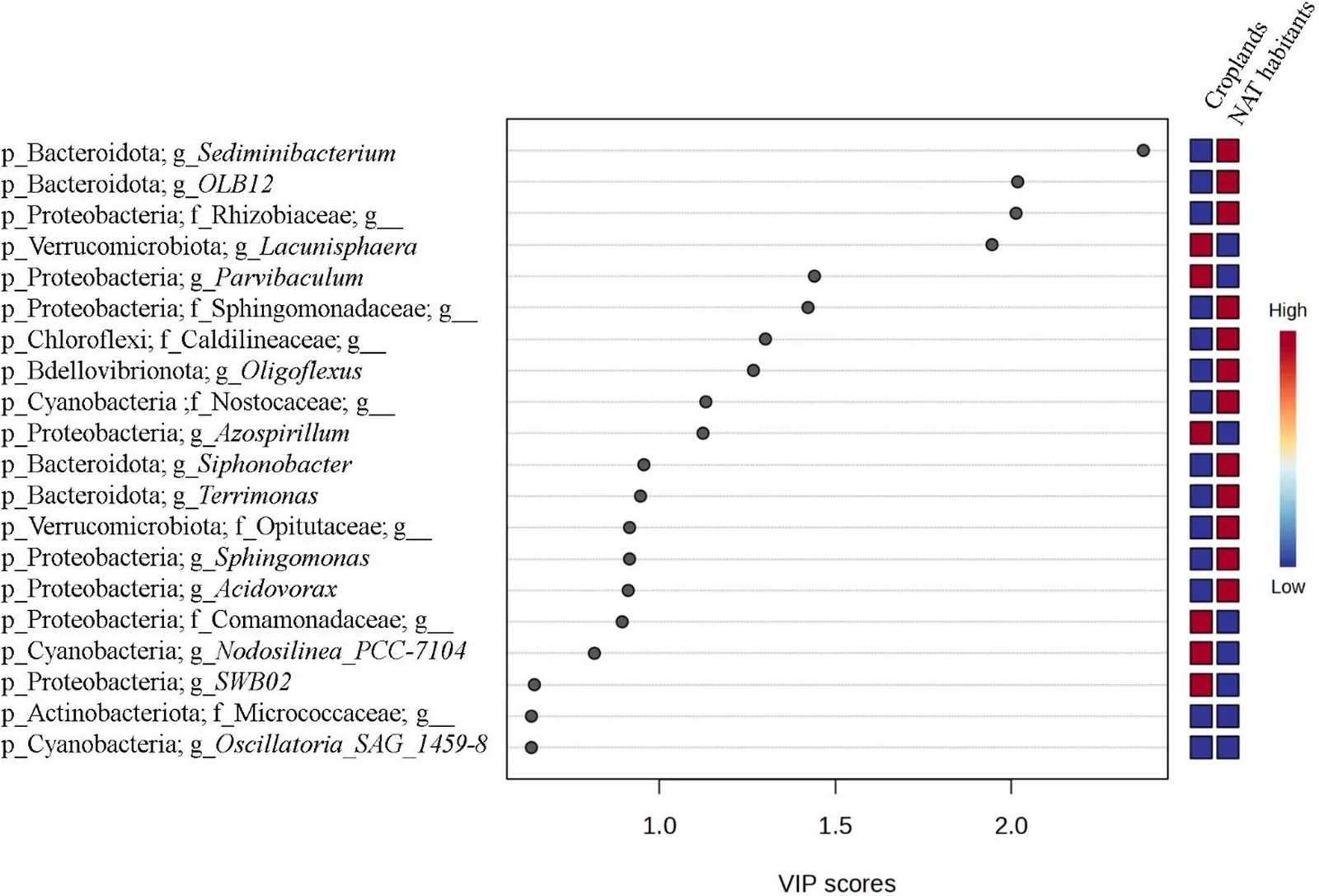
Partial Least Squares Discriminant Analysis (PLS-DA) of cyanobacteria-associated bacterial communities at the genus level, indicating compositional differentiation between cropland soils and non-agricultural terrestrial habitats (NAT habitats). The model was optimized using 5-fold cross-validation based on the Q² performance metric, with a maximum of two components evaluated. The analysis focuses on the 20 bacterial genera with the highest Variable Importance in Projection (VIP) scores, indicating their relative contribution to land-use-related differences in community composition. The heatmap represents relative representation patterns across treatments and is provided for visualization purposes; taxa were selected based on their VIP scores, indicating their contribution to group discrimination

PLS-DA analysis focusing on land-use categories revealed compositional differentiation between cropland soils and NAT habitats, driven by a subset of 20 bacterial genera with the highest VIP scores (Fig. 5). Several Bacteroidota-affiliated genera, including *Sediminibacterium*, *OLB12*, *Siphonobacter*, and *Terrimonas*, contributed to the discrimination of NAT-associated communities. A similar pattern was observed for themembers of Verrucomicrobiota, such as *Lacunisphaera* and uncultured representatives of the *Opitutaceae* in NAT habitats.

In contrast, multiple Proteobacteria, including *Parvibaculum*, *Sphingomonas*, *Azospirillum*, *Acidovorax*, and uncultured genera affiliated with *Rhizobiaceae* and *Comamonadaceae*,* c*ontributed more strongly to the separation of cropland-derived communities. Additional discriminatory taxa included *Oligoflexus* (Bdellovibrionota), uncultured members of *Caldilineaceae* (Chloroflexi), and uncultured representatives of *Micrococcaceae* (Actinobacteriota). Cyanobacterial taxa also contributed to land-use discrimination, with Nostocaceae*-*associated ASVs contributing to NAT-associated assemblages and Oscillatoriales-affiliated genera (*Nodosilinea* and *Oscillatoria*) contributing to cropland-associated assemblages. As in Fig. 4, the heatmap shows the relative representation of patterns across treatments and serves as a visual complement to VIP-based discrimination.

## Discussion

The absence of significant differences in alpha diversity between land-use types and sampling seasons indicated that functional selection imposed by cultivation constrained within-sample diversity. Because the analyses targeted a selective subset of cultivable cyanobacteria and associated bacteria rather than entire soil microbial communities, ecological differences among habitats and seasons more likely reflected shifts in community composition than overall diversity levels. Despite this apparent stability in diversity, strong and consistent compositional shifts were observed across both seasons and land-use types. The relatively modest proportion of variance explained by the first ordination axes is not unusual for amplicon-based microbial community datasets, where ecological variation is distributed across many taxa and environmental factors. In such high-dimensional datasets, the first components often capture only a fraction of the total variability, while statistically significant patterns of community differentiation remain detectable through multivariate tests such as PERMANOVA [18]. These findings indicated that environmental signals are primarily encoded in the relative representation of taxa within cyanobacteria-dominated communities, rather than overall diversity changes. Importantly, both seasonal and land-use effects converged on a similar subset of bacterial genera, suggesting shared ecological mechanisms underlying these patterns. Reasonably, this lack of correspondence between overall diversity patterns and community compositional dynamics has been widely reported in soil and cyanobacteria-associated microbial systems, where environmental gradients primarily restructure the relative abundance of recurrent taxa, with alpha diversity remaining comparatively stable, particularly under selective or cultivation-based conditions [9, 19, 20].

An additional aspect highlighted by this study is the role of cultivation-based selection in enhancing the detectability of ecological signals. Previous attempts to characterize terrestrial cyanobacterial diversity using broader metagenomic approaches resulted in inconsistent patterns across sites and seasons, partly due to database limitations and the high complexity of soil microbial communities [21, 22]. Among cyanobacterial taxa, members affiliated with *Nostocaceae* contributed to the differentiation of NAT habitats. In contrast, *Oscillatoriales*-affiliated genera (e.g., *Nodosilinea* and *Oscillatoria*) contributed more strongly to the discrimination of cropland-derived assemblages. Because taxa were selected based on their Variable Importance in Projection (VIP) scores in the PLS-DA model, these patterns reflect their relative contribution to group separation rather than direct evidence of ecological dominance in the original soil environments. This suggested that selective cultivation, when combined with high-throughput sequencing, may function not only as a biological filter but also as a methodological tool to improve signal-to-noise ratios in ecological studies of complex microbial systems [23].

The patterns observed suggest that seasonal dynamics exert a stronger influence on cyanobacterial representation under nitrogen-free enrichment than land-use category. In Mediterranean environments, soil microbial communities experience pronounced seasonal variability driven by fluctuations in temperature and moisture associated with the transition from wet winters to warm, dry summers. Such seasonal shifts strongly influence microbial activity and community composition in Mediterranean soils [24, 25]. During spring, increasing soil moisture following winter precipitation together with moderate temperatures can stimulate rapid microbial activation and competitive interactions among heterotrophic bacteria, potentially reducing the relative representation of cyanobacteria in enrichment cultures. The observed spring decline, therefore, likely reflects responses of cyanobacterial populations originating from light-exposed and soil-associated habitats, where seasonal variability in temperature and moisture can drive pronounced shifts in microbial community composition and ecological turnover [2, 26]. In contrast, some NAT environments included in this study, such as cave habitats, are characterized by relatively stable temperature and light conditions throughout the year and are therefore less affected by surface-driven seasonal fluctuations. Consequently, these sites are unlikely to be the primary drivers of the seasonal patterns observed in the enrichment cultures.

It should also be noted that all enrichment cultures were incubated at a constant temperature of 25 °C. While this standardized condition ensured comparability among samples, it may have favored taxa adapted to moderate temperatures and potentially underrepresented microorganisms adapted to cooler early-spring soil conditions. The relatively small difference between cropland and NAT habitats, averaged across seasons, further supports the interpretation that seasonal dynamics play a stronger role than land use in structuring these enrichment-derived assemblages.

Seasonal differentiation was driven mainly by changes in cyanobacteria-associated heterotrophic bacteria rather than by turnover of cyanobacterial taxa themselves. Genera such as *Flavobacterium*, *Pedobacter*, and *Terrimonas* are widely recognized for their ability to degrade complex organic substrates, including extracellular polysaccharides released by phototrophic organisms [27–29]. Seasonal variation in temperature, light availability, and moisture can influence cyanobacterial physiology and exudate production, thereby indirectly restructuring the associated heterotrophic community [30, 31].

Land-use-related differentiation followed a comparable pattern but likely reflected additional ecological filters. Cropland soils experience recurrent disturbance and fertilization history, whereas NAT habitats encompass more stable but heterogeneous environmental conditions. The observed shifts in genera such as *Parvibaculum*, *Rhodobacter*, and *Sphingomonas* across land-use types are consistent with their metabolic versatility and capacity to exploit diverse carbon sources when substrates undergo minimal processing, germination, or environmental exposure. These taxa are frequently reported in association with phototrophic hosts and are known to respond to changes in substrate availability and redox conditions [32–34].

The identification of *Azospirillum* and uncultured members of *Rhizobiales* among the genera contributing most strongly to seasonal and land-use discrimination highlights the strong selective pressure imposed by nitrogen-free enrichment conditions. Under nitrogen-free enrichment conditions, taxa capable of nitrogen fixation or efficient nitrogen scavenging may gain a competitive advantage through close metabolic coupling with cyanobacteria [35]. While *Nostocales* themselves are diazotrophic and therefore able to fix atmospheric nitrogen independently, heterotrophic diazotrophs are reported in association with phototrophic microorganisms within mixed microbial consortia [1]. In this context, their occurrence in the enrichment cultures likely reflects co-selection of functionally compatible microorganisms under nitrogen-limited cultivation rather than a strict metabolic dependence of cyanobacteria on these taxa.

At the same time, filamentous cyanobacteria such as *Nodosilinea* showed relative compositional stability across seasons and land-use types, suggesting that these taxa may represent a structurally stable component of the enrichment-derived communities. Such stability is consistent with previous observations indicating that many soil cyanobacteria show high ecological resilience and tolerance to environmental fluctuations. In cyanobacteria-centered microbial consortia, phototrophic taxa often provide a relatively stable ecological framework, whereas associated heterotrophic bacteria tend to respond more dynamically to environmental variation [36, 37].

Several limitations of this study should be acknowledged. The controlled incubation conditions, including a constant temperature of 25 °C, may have selectively favored the growth of certain taxa while underrepresenting microorganisms adapted to other environmental conditions, particularly those typical of early spring soils. In addition, the multivariate approaches employed, including PLS-DA, identify taxa contributing to group discrimination but do not represent formal tests of differential abundance or direct ecological interactions. Accordingly, the observed patterns should be interpreted as reflecting compositional shifts within functionally selected communities, rather than as direct evidence of ecological dominance or causal relationships in natural soil systems.

## Conclusions

These results indicated that, under nitrogen-free enrichment conditions, seasonal dynamics and land-use differences are primarily reflected in the reorganization of a limited set of cyanobacteria-associated bacterial genera rather than in changes in cyanobacterial diversity or dominance. Within these functionally selected systems, cyanobacteria appeared to form a relatively stable structural component, while associated heterotrophic bacteria respond more sensitively to environmental context, encoding seasonal and land-use signals in community composition.

Overall, this study highlighted the potential of selective cultivation approaches combined with high-throughput sequencing to reveal environmentally responsive patterns within complex microbial assemblages. However, the observed patterns should be interpreted in the context of enrichment-derived communities and do not necessarily reflect in situ microbial community structure.

## Supplementary Information

Below is the link to the electronic supplementary material.

Supplementary Material 1 (DOCX 721 KB)

## Data Availability

Raw sequencing data have been deposited in the NCBI Sequence Read Archive under BioProject accession number PRJNA1438445.
