# Supplementary material for: Land use and Season Drive Compositional Shifts in Cyanobacteria-dominated Soil Bacterial Communities
Source: Microb Ecol. 2026 Apr 23;89(1):121. doi: 10.1007/s00248-026-02759-6 (PMC13236745; doi:10.1007/s00248-026-02759-6)
Supplement: Supplementary file 1 — Supplementary Material 1 (DOCX 721 KB) [file 248_2026_2759_MOESM1_ESM.docx]

| **Supplementary Table S1.** Geographic coordinates and site description of natural and semi-natural (NAT) sampling habitats in southern Italy. NAT habitats included forest, prairie, and urban stone surface environments (city walls and pavements), as well as cave systems. Forest sites were located in the Mercadante Forest area, prairie sites in the Alta Murgia National Park, and urban stone surface sites within the city of Bari and surrounding areas. Latitude (N) and longitude (E) are reported in decimal degrees. | | | |
| --- | --- | --- | --- |
| Habitant | Site | Lat N | Long E |
| Mercadante Forest | Iazzo Nuovo | 40.870588 | 16.713185 |
|  | Bivio della Roverella | 40.875833 | 16.708333 |
|  | Cisterna Grottillo | 40.883611 | 16.690833 |
|  | Bitonto Wood | 40.975277 | 16.561944 |
| Alta Murgia Prairies | San Magno | 41.017593 | 16.377864 |
| Walls and pavements | Serra Mezzana | 40.913355 | 16.497448 |
|  | Torre di Nebbia | 41.037579 | 16.316326 |
|  | Ceglie del Campo | 41.063424 | 16.869026 |
|  | Via Conte Giusso | 41.093794 | 16.884761 |
|  | La Fitta Station | 41.061425 | 16.871510 |

| **Supplementary Table S2.** Sequencing statistics for all samples, including raw read counts, reads retained after denoising, and chimeric sequence counts. Reads retained (%) indicates the proportion of reads remaining after denoising relative to raw reads. | | | | | | | | | | | |
| --- | --- | --- | --- | --- | --- | --- | --- | --- | --- | --- | --- |
| **sample-id** | **Season** | **Land use** | **reads-raw** | **unique-reads-derep** | **reads-derep** | **unique-reads-deblur** | **reads-deblur** | **unique-reads-chimeric** | **reads-chimeric** | **unique-reads-hit-reference** | **reads-hit-reference** |
| sample-1 | Spring | NAT habitants | 55154 | 264 | 994 | 115 | 379 | 7 | 8 | 19 | 206 |
| sample-2 | Spring | Cropland | 57889 | 206 | 922 | 63 | 318 | 5 | 6 | 8 | 198 |
| sample-3 | Spring | Cropland | 65924 | 304 | 1278 | 106 | 436 | 5 | 6 | 11 | 247 |
| sample-4 | Spring | Cropland | 43367 | 149 | 521 | 70 | 176 | 0 | 0 | 9 | 69 |
| sample-5 | Spring | NAT habitants | 94690 | 381 | 1369 | 225 | 642 | 21 | 24 | 20 | 263 |
| sample-6 | Spring | Cropland | 90576 | 301 | 1115 | 95 | 367 | 6 | 10 | 13 | 186 |
| sample-7 | Spring | Cropland | 72547 | 186 | 711 | 72 | 268 | 5 | 7 | 15 | 147 |
| sample-8 | Spring | NAT habitants | 81423 | 257 | 831 | 181 | 453 | 13 | 16 | 16 | 116 |
| sample-9 | Spring | NAT habitants | 60847 | 198 | 739 | 133 | 361 | 10 | 11 | 18 | 157 |
| sample-10 | Spring | NAT habitants | 61171 | 255 | 1126 | 85 | 423 | 5 | 6 | 13 | 266 |
| sample-11 | Autumn | NAT habitants | 68650 | 269 | 1019 | 98 | 342 | 6 | 8 | 20 | 193 |
| sample-12 | Autumn | Cropland | 71175 | 269 | 1000 | 69 | 279 | 0 | 0 | 15 | 184 |
| sample-13 | Autumn | Cropland | 77980 | 250 | 909 | 96 | 293 | 6 | 6 | 11 | 111 |
| sample-14 | Autumn | Cropland | 71231 | 254 | 916 | 103 | 313 | 7 | 8 | 16 | 140 |
| sample-15 | Autumn | NAT habitants | 78032 | 290 | 1199 | 95 | 417 | 10 | 15 | 16 | 236 |
| sample-16 | Autumn | Cropland | 46086 | 104 | 302 | 66 | 137 | 8 | 9 | 7 | 44 |
| sample-17 | Autumn | Cropland | 37140 | 95 | 314 | 60 | 134 | 5 | 5 | 5 | 39 |
| sample-18 | Autumn | NAT habitants | 69839 | 323 | 1218 | 136 | 403 | 14 | 15 | 24 | 217 |
| sample-19 | Autumn | NAT habitants | 92689 | 233 | 734 | 170 | 398 | 11 | 14 | 12 | 88 |
| sample-20 | Autumn | NAT habitants | 68077 | 302 | 1192 | 118 | 414 | 14 | 14 | 24 | 241 |


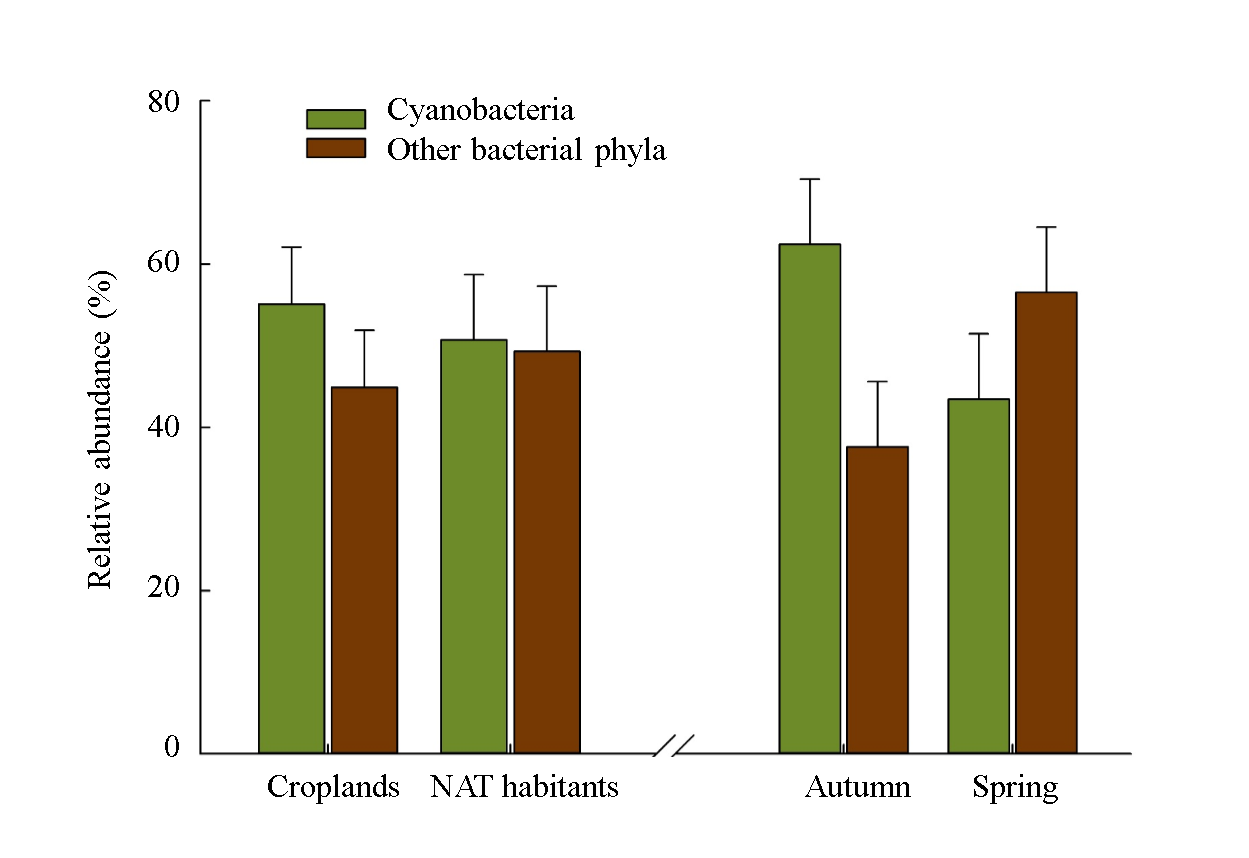


**Supplementary Fig. 1.** Relative abundance of cyanobacteria and other bacterial phyla in nitrogen-free enrichment cultures summarized by land-use category and season. Values represent phylum-level relative abundances averaged across samples.


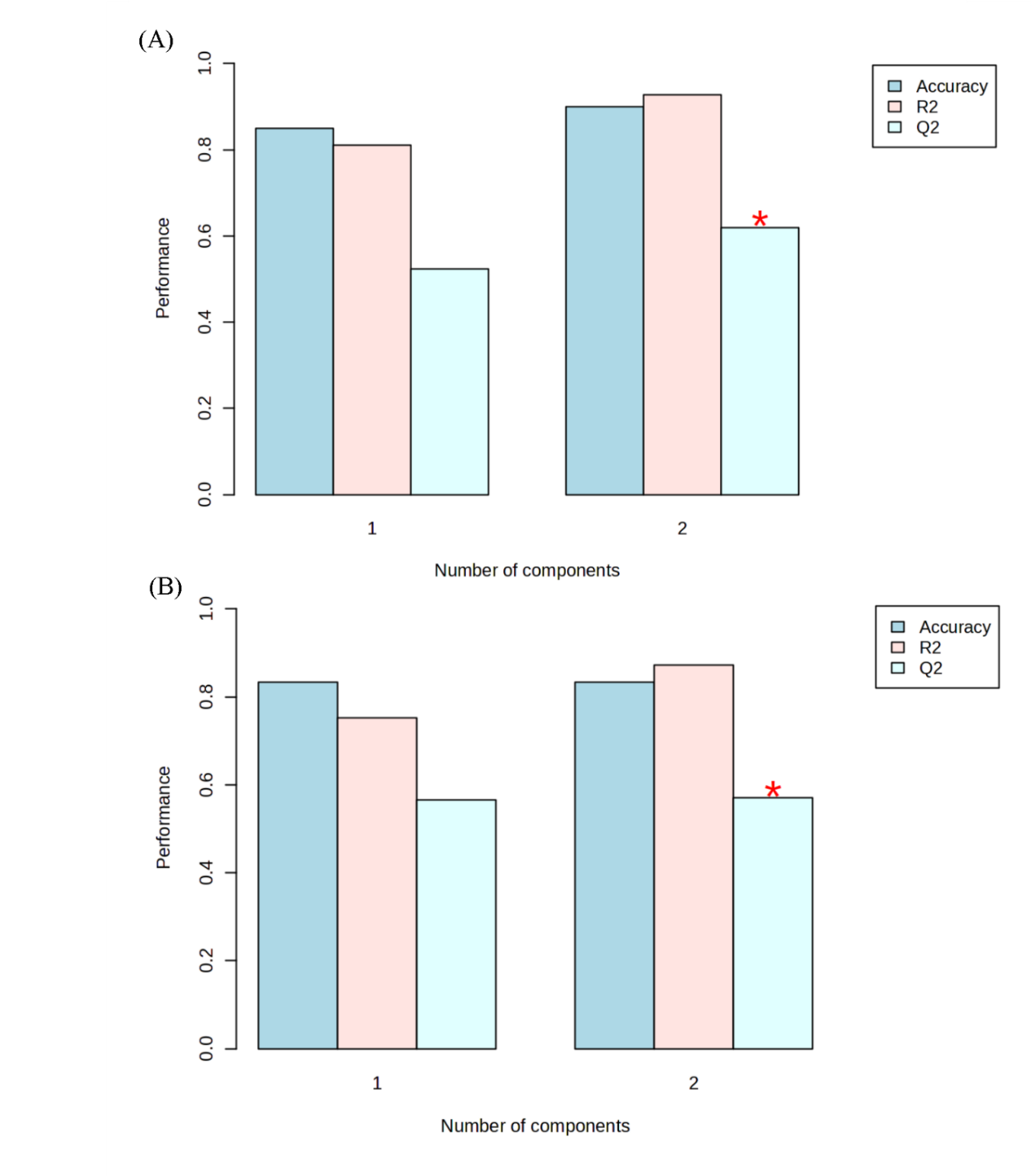


**Supplementary Fig. 2.** Performance metrics of the Partial Least Squares Discriminant Analysis (PLS-DA) model based on different numbers of components. Panel **(A)** shows model validation for seasonal discrimination, and panel **(B)** for land-use discrimination. Bars represent classification accuracy, goodness-of-fit (R²), and predictive ability (Q²) calculated using 5-fold cross-validation. R² indicates the proportion of variance explained by the model, whereas Q² reflects its predictive performance estimated from cross-validated prediction errors. The models indicated good explanatory power and reliable predictive capability without evidence of overfitting.
